# Blockade of Erythropoietin-Producing Human Hepatocellular Carcinoma Receptor B1 in Spinal Dorsal Horn Alleviates Visceral Pain in Rats

**DOI:** 10.1155/2021/7582494

**Published:** 2021-04-07

**Authors:** Chen-Li Sun, Cheng-Wen Li, Nong He, Yuan-Zhang Tang, Xiu-Liang Li, Fu-Shan Xue, Jia-Xiang Ni

**Affiliations:** ^1^Department of Pain Management, Xuanwu Hospital of Capital Medical University, Beijing, China; ^2^Department of Anesthesiology, Beijing Friendship Hospital, Capital Medical University, Beijing, China

## Abstract

**Objective:**

This experiment was designed to determine whether erythropoietin-producing human hepatocellular carcinoma (Eph) receptors were involved in the development of visceral pain.

**Methods:**

Adult male Sprague-Dawley rats were randomly divided into three groups receiving different treatments (*n* = 16 per group): intracolonic vehicle (control group), intracolonic 2, 4, 6-trinitrobenzene sulfonic acid (TNBS) (TNBS group), and intracolonic TNBS and intrathecal EphB1 receptor blocking reagent (TNBS + EphB2-Fc group). Visceral hyperalgesia was evaluated with quantification of visceral pain threshold induced by colorectal distention. The spinal expressions of EphB1 and ephrinB2 and levels of their phosphorylated forms (p-EphB1 and p-ephrinB2) were assessed by Western blotting and immunohistochemistry.

**Results:**

The TNBS-treated rats developed significant visceral hyperalgesia. The spinal expressions of EphB1, p-EphB1, ephrinB2, and p-ephrinB2 were significantly increased in the TNBS group compared with the control group, but visceral hyperalgesia and elevation of spinal EphB1 and p-EphB1 expressions were evidently alleviated by intrathecal administration of EphB2-Fc in the TNBS + EphB2-Fc group. The number of EphB1- and p-EphB1-immunopositive cells, the average optical (AO) value of EphB1, and its phosphorylated form in the spinal dorsal horn were significantly increased in the TNBS group than in the control group, but they were obviously reduced by intrathecal administration of EphB2-Fc. There were no significant differences in the number of ephrinB2- and p-ephrinB2-immunopositive cells and the AO value of ephrinB2 and its phosphorylated form between the TNBS and TNBS + EphB2-Fc groups.

**Conclusion:**

EphB1 receptors in the spinal dorsal horn play a pivotal role in the development of visceral pain and may be considered as a potential target for the treatment of visceral pain.

## 1. Introduction

Abdominal pain is one of the major and troublesome hallmarks of gastrointestinal diseases, such as gastrointestinal tumors, inflammatory bowel disease, and irritable bowel syndrome. Visceral hyperalgesia, which refers to a decrease of visceral pain threshold to mechanical distension, is regarded as one of the pivotal players in the development of abdominal pain [1–3]. Increasing research has attempted to reveal the molecular mechanism of visceral hyperalgesia [4–8], but this issue is still not completely understood.

Erythropoietin-producing human hepatocellular carcinoma (Eph) receptors, including types A (A1–A10) and B (B1–B6), are the largest subfamily of transmembrane receptor tyrosine kinases (RTKs). It has been shown that the Eph receptors and their ligands Eph-receptor interacting proteins (ephrin) play a critical role in the development of the nervous system, such as regulation of axon guidance [9, 10]. Currently, it has been known that ephrin ligands have two types: glycosylphosphatidylinositol (GPI) anchored type (ephrinA1–A10) and transmembrane type (ephrinB1–B3). Recently, accumulating evidence indicates that the ephrinB/EphB signaling pathway is a crucial player in the development of various chronic pains, including inflammatory pain, neuropathic pain, bone cancer pain, and viscerovisceral referred pain [11–20]. This signaling pathway can not only regulate the function of synapse [21–26] but also modulate chronic pain by interacting with N-methyl-D-aspartate (NMDA) receptors [20, 26–28] as well as other targets [29–31]. However, the importance of this signaling pathway is mainly identified in the development of somatic pain. Up to now, only a few studies are focused on the involvement of ephrinB2/EphB1 signaling pathway in the pathogenesis of visceral pain [32, 33]. For example, a previous work using pelvic-urethra-related pain model found that ephrinB2 potentiated the pelvic-urethra reflex due to phosphorylation of EphB1 and/or 2 via the Src kinase [32]. Recently, another study using ephrinB2 knockout animals demonstrated that both ephrinB2 and EphB1 played important roles in 2, 4, 6-trinitrobenzene sulfonic acid- (TNBS-) induced chronic (postinflammatory) visceral pain, but only ephrinB2 was involved in the development of stress-induced visceral pain [33]. Most important, it remains unclear whether administration of specific blockers to interrupt the phosphorylation of EphB1 and/or expression of ephrinB2 would attenuate visceral pain. Thus, this experiment was designed to assess the spinal expressions of EphB1 and ephrinB2 and their phosphorylation levels in a rat model of visceral pain induced by intracolonic injection of TNBS and determine the effects of using an EphB1 receptor blocking reagent on the development of visceral pain and the spinal expression of EphB1 receptors.

## 2. Data and Methods

### 2.1. Animals

After the experimental protocol was approved by the Animal Care and Use Committee of Xuanwu Hospital of Capital Medical University, adult male Sprague-Dawley (SD) rats, aged 8–10 weeks and weighing 150–250 g, were used in this experiment. Furthermore, all animal experiments were conducted in the Animal Experiment Center of Xuanwu Hospital. The animals were housed in plastic cages with soft bedding at room temperature and a 12 : 12-hour light-dark cycle every day. They were free to access food and water and acclimated for 3 days before the experiment. By using a randomized digital table, the rats were divided into three groups receiving different treatments (*n* = 16 per group): intracolonic vehicle (control group), intracolonic TNBS (TNBS group), and intracolonic TNBS and intrathecal EphB1 receptor blocking reagent (TNBS + EphB2-Fc group).

### 2.2. Establishment of Colonic Hyperalgesia Model

In this experiment, a colonic hyperalgesia model was established by injection of TNBS (Sigma-Aldrich, MO, USA), as previously described [34, 35]. Briefly, the rats were anesthetized with 2% isoflurane and fasten in a supine position. A polyethylene catheter (1.2 mm in diameter) was inserted into the colon, with the distal tip 6 cm far from the anus. The TNBS solution of 100 *μ*l (14–16 mg/ml in 25% ethanol) was injected into the colon via the catheter in the TNBS and TNBS + EphB2-Fc groups, and vehicle solution of 100 *μ*l including only 25% ethanol was injected in the control group. Then, the lower portion of the rat body was elevated for 30 seconds.

### 2.3. Histological Assessment of Colonic Damage

The level of colonic damage was evaluated before administration of TNBS (baseline), 1 day after administration of TNBS (inflammatory stage) and 15 days after administration of TNBS (postinflammatory stage). Four animals were euthanized under anesthesia with 2% isoflurane at each time-point. The 1 cm distal colon was excised, rinsed with saline, fixed in 4% formalin, and embedded in paraffin. Sections of full-thickness colon samples (8 *μ*m thick) were stained with hematoxylin (Leica Biosystems) and eosin (Leica Biosystems) and observed under microscopy. The levels of colonic damage were scored according to the criteria described in previous literature [36, 37]: mucosal architecture loss (0–3); goblet cell depletion (0, absent; 1, present); crypt abscess (0, absent; 1, present); cellular infiltration (0–3); and tunica muscularis thickening (0–3).

### 2.4. EphB2-Fc Preparation and Intrathecal Injection

This experiment used mouse recombinant chimaera of EphB2-Fc (Bio-Techne, MN, USA) as an EphB1 receptor blocking reagent, as reported in previous studies with rats [17, 18, 24]. By combining with the endogenous ephrinB, EphB2-Fc can cause EphB1 substituted and cleaved, and then result in the blockade of the downstream signals of EphB1. EphB2-Fc chimaera was prepared on the day of intrathecal injection (100 *μ*g/ml in sterile PBS). On 14, 15, and 16 days after intracolonic injection of TNBS or vehicle, the rat was anesthetized with 2% isoflurane and lumbar puncture was performed at L4-5 intervertebral space. When successful intrathecal puncture was confirmed by a typical tail-flick, EphB2-Fc of 5 *μ*l was administrated in the TNBS + EphB2-Fc group and vehicle (sterile PBS) of 5 *μ*l in the other groups, once a day for three consecutive days.

### 2.5. Behavioral Test

In each animal, colorectal distension (CRD) was carried out 17 days after intracolonic injection of TNBS or vehicle, as previously described [4, 33, 38]. In brief, a latex double-lumen catheter was attached to a balloon dilator with a diameter of 5 mm. The lubricated dilator was gently inserted into the descending colon until its distal tip was 6 cm from the anus. Then, CRD was maintained by injection of increasing air (0.1–5.0 ml). The rats were placed in a small lucite cubicle in which they were kept waking up and acclimated for 30 min before CRD.

The abdominal withdrawal reflex (AWR) responses, which referred to a sudden and persistent abdominal muscle contraction with abdomen lift off the platform, were evaluated by an observer who was blind to the group assignment. The volume of injected air that elicited an observable AWR was used to quantify the visceral pain threshold. For each rat, the behavioral test was performed three times with an interval of 5 min. The averaged value of three measurements was used as the visceral pain threshold.

### 2.6. Western Blotting

After the behavioral test, animals were euthanized under anesthesia with 2% isoflurane, and then the spinal cord at lumbosacral levels (L6-S1) was freshly extracted and stored in liquid nitrogen. To quantify the expression levels of EphB1, ephrinB2, and their phosphorylated forms (p-EphB1 and p-ephrinB2) in the spinal cord by Western blotting, spinal samples were homogenized in ice-cold (4°C) lysis buffer containing 50 mM Tris-HCl, 150 mM NaCl, 1% Triton X-100, 0.5% deoxycholate, 0.1% SDS, 0.2 mM EDTA, 10 mM NaF, 10 *μ*g/ml aprotinin, 1 *μ*g/ml leupeptin, 10 *μ*g/ml pepstatin, 0.4 mM 4-(2-amino-ethyl)-benzenesulfonyl fluoride, and 1 mM sodium orthovanadate (pH 7.5). Next, precipitation procedures were conducted on the homogenate. The protein concentrations were analyzed using a BCA protein assay kit (Pierce Biotechnology, MA, USA). These proteins were separated with 8% or 10% SDS-PAGE and then transferred to a nitrocellulose membrane.

The protein levels were detected by antibodies including anti-EphB1 (Santa Cruz Technology, CA, USA), anti-phosphorylated EphB1 (Santa), anti-ephrinB2 (Bioss Biotechnology, MA, USA), and anti-phosphorylated ephrinB2 (Bioss) antibodies. The membrane was blocked with 5% milk in Tris-buffered saline for 1 h, incubated with anti-phosphorylated EphB1 (1 : 1000) and anti-phosphorylated ephrinB2 (1 : 1000) antibodies, and then incubated with horseradish peroxidase-conjugated secondary antibody. The bands were processed by enhanced chemiluminescence reagents (Millipore, MA, USA). The same membrane was stripped and processed with primary antibodies against EphB1 (1 : 1000) and ephrinB2 (1 : 1000).

The films were digitized, and densitometric quantification of immunoreactive bands was performed by using the Image *J* 1.51 software (Softonic International, Barcelona, Spain). The expression of a specific protein was normalized to that of *β*-tubulin.

### 2.7. Immunohistochemistry

The lumbosacral spinal cord sample was fixed in 4% formalin and embedded in paraffin. Sections 8 *μ*m thick were incubated with polyclonal rabbit antibodies of anti-EphB1 (1 : 200, Santa), anti-ephrinB2 (1 : 200, Santa), anti-phosphorylated EphB1 (1 : 200, Santa), and anti-phosphorylated ephrinB2 (1 : 200, Santa). The anti-rabbit IgG antibody (1 : 200, Sigma) was used to incubate the tissue sections. After the tissue slides were incubated with ABC reagent (1 : 200, Thermo Fisher Scientific), they were strained with diaminobenzidine solution for 1 to 2 min and then rinsed in distilled H_2_O_2_. Next, the tissue slides were strained with hematoxylin solution for 1 to 2 min and were then rinsed in distilled H_2_O_2_ again. Normal goat serum was used as a negative background control. Finally, EphB1 and ephrinB2 immunopositive cells in the spinal dorsal horn were counted and the integrated optical density values were measured by the IPP 6.0 software (Media Cybernetics). Averaged optical (AO) values were calculated by the ratio of integrated optical density/area of tissue.

### 2.8. Statistical Analysis

All data are expressed as the mean ± standard error of mean (SEM). The differences in the spinal expression levels of detected proteins and the threshold of visceral pain among groups were analyzed using one-way analysis of variance (ANOVA) with the SPSS software (Version 20.0, International Business Machines Corporation, NY, USA). A *P* value of less than 0.05 was considered statistically significant.

## 3. Results

### 3.1. Colonic Damage

**Figure 1:**
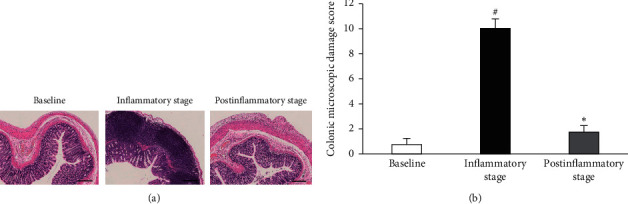
(a): The typical photomicrographs of colonic HE staining before administration of TNBS (baseline), 1 day after administration of TNBS (inflammatory stage), and 15 days after administration of TNBS (postinflammatory stage); (b): comparisons of colonic microscopic damage scores among three time-points. *n* = 4 in each group. Data are expressed as the mean ± SEM. ^#^*P* < 0.05 versus Baseline. ^*∗*^*P* < 0.05 versus inflammatory stage. Scale bar = 250 *μ*m.

As shown in Figure 1, microscopic scores of colonic damage were markedly increased at 1 day after administration of TNBS compared with those at the baseline. However, microscopic scores of colonic damage were significantly decreased at 15 days after administration of TNBS compared with those at 1 day after administration of TNBS. Furthermore, there was no difference in the microscopic scores of colonic damage between the baseline and 15 days after administration of TNBS.

### 3.2. Behavioral Test

**Figure 2:**
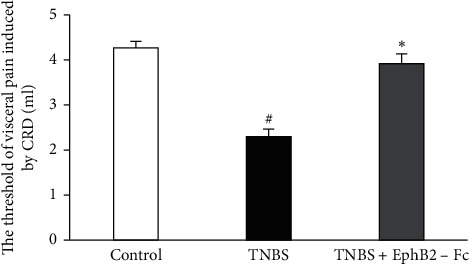
The comparisons of visceral pain thresholds induced by the colorectal distension among three groups. (*n*) = 8 in each group. Data are expressed as the mean ± SEM. ^#^*P* < 0.05 versus the control group. ^*∗*^*P* < 0.05 versus TNBS group.

As shown in Figure 2, the visceral pain threshold was significantly decreased in the TNBS group compared with the control group. Intrathecal administration of EphB2-Fc evidently attenuated the TNBS-induced visceral hyperalgesia.

### 3.3. Spinal Expressions of EhpB1, ephrinB2, p-EphB1, and p-ephrinB2

**Figure 3:**
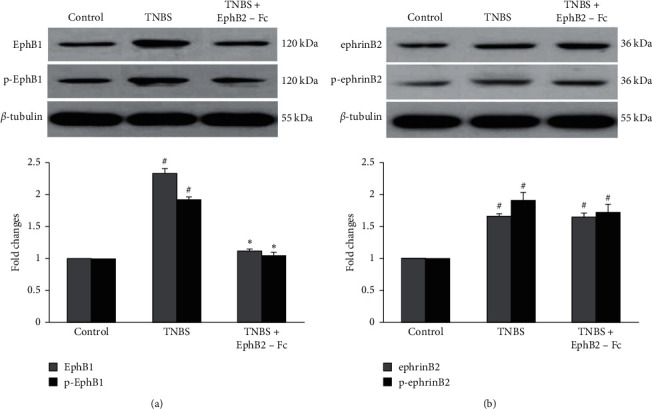
The expression levels of spinal EphB1 and p-EphB1 (a), and ephrinB2 and p-ephrinB2. (b) The upper parts of pictures are the representative Western blot bands and the lower parts are comparisons of their expression levels among three groups. (*n*) = 8 in each group. Data are expressed as the mean ± SEM. ^#^*P* < 0.05 versus control group. ^*∗*^*P* < 0.05 versus TNBS group.

As compared with the control group, spinal expression levels of EphB1 and p-EphB1 were markedly elevated in the TNBS group, and these expression evaluations were significantly decreased by intrathecal administration of EphB2-Fc in the TNBS + EphB2-Fc group (Figure 3(a)).

As compared with the control group, the spinal expression level of ephrinB2 was significantly increased in the TNBS group, with a remarkable upregulation of p-ephrinB2 expression. However, intrathecal administration of EphB2-Fc failed to suppress the TNBS-induced expression upregulation of two proteins (Figure 3(b)).

### 3.4. Immunohistochemical Assay for EhpB1, ephrinB2, p-EphB1, and p-ephrinB2 in the Spinal Dorsal Horn

**Figure 4:**
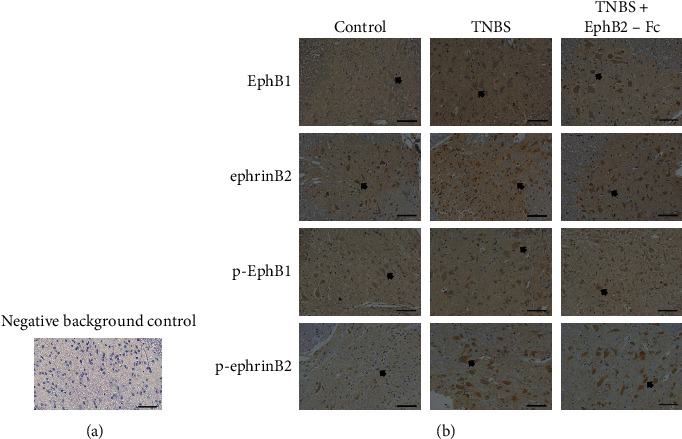
(a): The typical photomicrographs of immunostaining for negative background control; (B) EphB1, ephrinB2, and their phosphorylated forms in the spinal dorsal horns of three groups (b). Arrows indicate representative EphB1-, ephrinB2-, p-EphB1-, or p-ephrinB2-immunopositive cells. Scale bar = 50 *μ*m.

When goat serum was used instead of the first antibody for a negative background control, no immunopositive cell was noted (Figure 4(a)). The representative immunohistochemical staining images of EphB1, ephrinB2, and their phosphorylated forms distribution in the spinal dorsal horn are shown in Figure 4(b). The expression levels of these proteins were obviously increased in the TNBS group compared to the control group. The expression levels of EphB1 and p-EphB1 were markedly reduced in the TNBS + EphB2-Fc group compared to the TNBS group, but expression levels of ephrinB2 and p-ephrinB2 were not different between TNBS and TNBS + EphB2-Fc groups.

**Figure 5:**
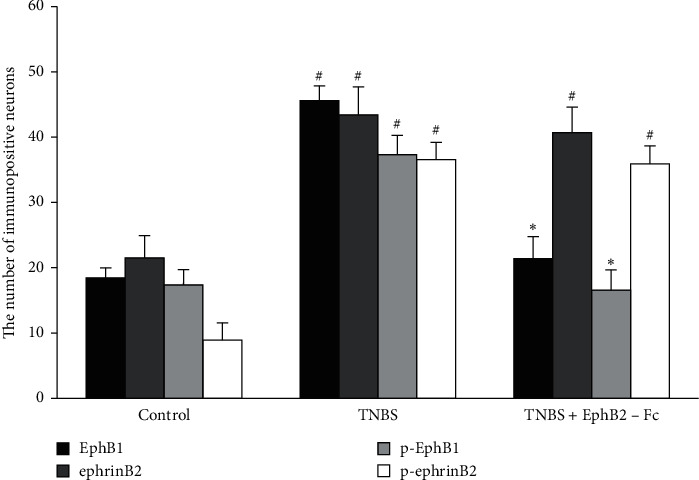
The comparisons for the numbers of EphB1-, ephrinB2-, p-EphB1-, and p-ephrinB2-immunopositive cells in the spinal dorsal horn among three groups. (*n*) = 4 in each group. Data are expressed as the mean ± SEM. ^#^*P* < 0.05 versus the control group. ^*∗*^*P* < 0.05 versus the TNBS group.

The numbers of EphB1- and p-EphB1-immunopositive cells in the spinal dorsal horn were significantly increased in the TNBS group compared to the control group, but they were obviously decreased by intrathecal administration of EphB2-Fc. The numbers of ephrinB2- and p-ephrinB2-immunopositive cells were significantly increased in the TNBS group compared with the control group. There was no significant difference in the numbers of ephrinB2- and p-ephrinB2-immunopositive cells between TNBS and TNBS + EphB2-Fc groups (Figure 5).

**Figure 6:**
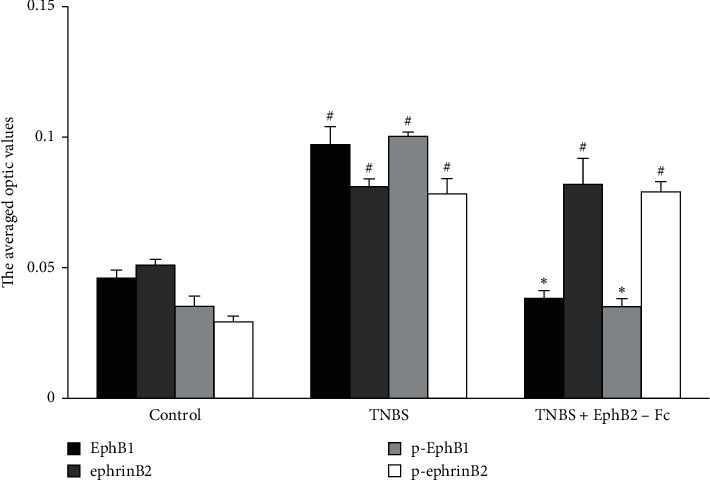
The comparisons for the averaged optic values of EphB1, ephrinB2, and their phosphorylated forms in the spinal dorsal horn among three groups. *n* = 4 in each group. Data are expressed as the mean ± SEM. ^#^*P* < 0.05 versus the control group. ^*∗*^*P* < 0.05 versus the TNBS group.

The AO values of EphB1, ephrinB2, and their phosphorylated forms in the spinal dorsal horn are shown in Figure 6. As compared to the control group, AO values of EphB1 and p-EphB1 were obviously elevated in the TNBS group. Intrathecal administration of EphB2-Fc significantly suppressed the TNBS-induced increase of AO values of EphB1 and p-EphB1. The AO values of ephrinB2 and p-ephrinB2 were obviously higher in the TNBS group than in the control group, but they were not decreased by intrathecal administration of EphB2-Fc.

## 4. Discussions

The present experiment aimed to assess the effects of EphB1 receptor blockade on the development of visceral hyperalgesia and spinal expressions of EphB1 and ephrinB2 in a rat model of visceral pain induced by TNBS. Our results showed that EphB1 receptor blockade with EphB2-Fc could significantly alleviate visceral hyperalgesia and decrease spinal expression of EphB1.

Accumulating evidence indicates that ephrinB/EphB signaling is a critical player in the development of somatic and neuropathic pain. The expression of EphB1 is upregulated and the nociceptive behaviors of thermal hyperalgesia and/or mechanical allodynia are induced by applying cutaneous inflammation [27], peripheral nerve injury [11, 14, 16, 24], or carcinoma cells inoculation [17, 18, 39]. In the neuropathic pain models evoked by chronic constriction nerve injury [12–14] and/or partial nerve ligation [13], however, these nociceptive behaviors can be alleviated by interrupting the EphB1 signaling pathway with EphB1 small interfering RNA [14] or knockout technique [12, 13]. Furthermore, blockade of EphB1 receptors has been demonstrated to produce a remarked alleviation on these nociceptive behaviors in the rodents evoked by chronic constriction nerve injury [12, 24, 29, 30], tumor cell implantation [18, 39], or subcutaneous remifentanil infusion [27, 31]. By using a visceral hyperalgesia model induced by intracolonic TNBS, this experiment showed that intrathecal administration of EphB1 receptor blocking reagent significantly alleviated visceral hyperalgesia with downregulation of spinal EphB1 expression. This is in accord with the results of a recent study [33], in which visceral hyperalgesia induced by intracolonic TNBS in wild-type mice did not occur in the ephrinB2 knockout mice.

Our results also indicated that intracolonic TNBS-provoked visceral hyperalgesia, with a significant upregulation of spinal p-EphB1 expression. The EphB1 receptor blocking reagent not only alleviated the upregulation of spinal EphB1 expression but also decreased the level of spinal p-EphB1 in the rats with TNBS-induced visceral pain. This suggested that phosphorylation of EphB1 receptors may play a critical role in the development of postinflammatory visceral hyperalgesia. Even previous studies demonstrated that EphB1 receptor agonist could induce transient thermal hyperalgesia in the rats [18, 24, 28, 29], with an increased phosphorylation level of NR2B (a subunit of NMDA receptors) in the spinal cord [18, 28], which was reversed via pretreatment of Src-family kinase inhibitor [28]. This transient thermal hyperalgesia was not related to the upregulation of total EphB1 but was dependent on the activation of EphB1, indicated by the upregulation of phosphorylated NR1 (a subunit of NMDA receptors) and NR2B [18]. Additionally, the pretreatment with NMDA receptor antagonist could eliminate thermal hyperalgesia and mechanical allodynia in the rats receiving an intrathecal administration of EphB1 receptor agonist (ephrinB1-Fc) [27]. Similarly, knockdown of ERK5 inhibited activation of cAMP-responsive element-binding protein (CREB) and alleviated thermal hyperalgesia and mechanical allodynia provoked by intrathecal administration of EphB1 receptor agonist (ephrinB2-Fc) [29]. These findings of previous studies demonstrate that NMDA receptors, Src-family kinase, and ERK/CREB may be the potential downstream signal transducers of EphB1 receptors, and the interactions between EphB1 receptor and its downstream signal transducers may be crucial for the development of hyperalgesia and allodynia induced by intrathecal administration of EphB1 receptor agonist. The results of the present experiment were similar to the findings from a previous study by Slack and colleagues [28], in which the pretreatment with EphB1 receptor blocking reagent attenuated thermal hyperalgesia and mechanical allodynia and inhibited the upregulation of phosphorylated NR2B expression in an inflammatory pain model induced by intraplantar injection of carrageenan. Furthermore, Peng and colleagues [20] reported that the pretreatment with Src inhibitor could reverse phosphorylation of Src and NR2B receptors and alleviate the urethra reflex sensitization in the rats of mustard oil-induced acute colitis. The same results have been also described in a previous study with the pretreatment using a NMDA receptor antagonist in the rats receiving intrathecal administration of ephrinB2 [32]. The pretreatment with EphB receptor block reagent can not only produce these effects achieved by the pretreatment with Src inhibitor or NMDA receptor antagonist but also reverse the phosphorylation of EphB1/2 [20, 32]. In addition, the pretreatment with a NMDA receptor antagonist has been demonstrated to eliminate thermal hyperalgesia and mechanical allodynia in the rats receiving intraplantar injection of remifentanil [27]. These previous findings suggest that both NMDA receptors and Src-family kinase are likely to be downstream signal transducers of EphB1 receptors, through which EphB1 receptors mediate in inflammatory pain and viscerovisceral referred pain.

As described by Klein [40], ephrin-Eph system can function in a bidirectional fashion, to trigger reverse signaling into the ephrin-expressing cells, forward signaling into the Eph-expressing cells, or bidirectional signaling into both cells; that is, Eph receptors can act as a ligand and an ephrin ligand can also play a receptor role. However, this experiment showed that EphB1 blocking reagent failed to reduce the upregulation of spinal ephrinB2 and p-ephrinB2 expressions in the TNBS-treated rats. This indicates that EphB1 blocking reagent maybe plays a forward role by inhibiting activation of related downstream receptors and kinases. Previous studies in the bone cancer pain model and CCI-induced neuropathic pain model demonstrated that EphB1 receptor antagonist relieved hyperalgesia and allodynia by downregulating phosphorylation of NR1, NR2B, Src, ERK, MK2, and CREB or reducing activation of ERK5/CREB in the spinal cord [18, 29]. Thus, similar signaling mechanisms may also be attributable to postinflammatory visceral hyperalgesia induced by TNBS in this experiment. In fact, EphB1 receptors have been identified in the spinal cord, locating in the neurons [11, 16–18], astrocytes, and microglial cells [17]. Thus, the detailed role of phosphorylation of spinal EphB1 receptors in the development of visceral pain warrants future study.

As a relay station for pain signals, the spinal dorsal horn has many signal transducers involving pain. In the bone cancer pain model induced by tumor cell implantation [17, 39] and the neuropathic pain model provoked by chronic constriction nerve injury [11] or crush spinal nerve injury [16], the immunostaining and/or immunofluorescence assessment shows that EphB1 receptors are mainly distributed in the spinal dorsal horn. In the rats with tumor cell implantation-evoked bone cancer pain by immunoblotting [39], moreover, ephrinB2 ligands have been observed to be mainly located in the spinal dorsal horn. In this experiment, thus, the numbers of EphB1, ephrinB2, p-EphB1, and p-ephrinB2 immunopositive cells in the dorsal horn and their AO values were assessed by immunohistochemistry to further determine the changes of their expressions in the spinal dorsal horn. Similar to our results obtained by the Western blotting, intrathecal administration of EphB1 receptor blocking reagent significantly attenuated visceral hyperalgesia, with a significant downregulation of EphB1 and p-EphB1 expressions in the spinal dorsal horn in the TNBS-treated rats. However, intrathecal administration of EphB1 blocking reagent failed to decrease the upregulation of ephrinB2 and p-ephrinB2 expressions in the spinal dorsal horn in the TNBS-treated rats. These results further support that the EphB1 receptors in the spinal dorsal horn play a pivotal role in the development of TNBS-induced visceral pain.

It must be pointed out that there are several limitations in the design of this experiment. First, as intrathecal administration of EphB1 receptor blocking reagent has shown an inability to change normal pain perception in the rats [18], a group of animals receiving EphB1 receptor blocking reagent on 14, 15, and 16 days after the intracolonic vehicle was not included. Second, as the behavioral test was not performed before the initial intervention, it was unable to compare the visceral pain thresholds before and after intervention. Third, this experiment was mainly focused on the role of the ephrinB2-EphB1 signaling pathway in the development of visceral pain. It cannot provide any clue regarding the possible contributions of the related downstream signaling pathways to the development of visceral hyperalgesia. Thus, further experiments are still needed to address these issues.

In conclusion, this experiment demonstrates that blockade of spinal EphB1 receptors is able to alleviate the visceral hyperalgesia and reduce upregulation of spinal EphB1 expression in a rat model of TNBS-provoked visceral pain. It indicates that spinal EphB1 receptors play a crucial role in the development of visceral pain and may be a promising target of visceral pain treatment.

## Data Availability

The data are available on request.

## Abbreviations

Eph: Erythropoietin-producing human hepatocellular carcinoma
RTKs: Receptor tyrosine kinases
EPHRIN: Eph-receptor interacting proteins
GPI: Glycosylphosphatidylinositol
NMDA: N-Methyl-D-aspartate
TNBS: 2, 4, 6-Trinitrobenzene sulfonic acid
SD: Sprague-Dawley
HE: Hematoxylin-eosin
CRD: Colorectal distension
AWR: Abdominal withdrawal reflex
AO: Averaged optical
SEM: Standard error of the mean
ANOVA: Analysis of variance
CREB: CAMP responsive element-binding protein.
